# New Episodic Learning Interferes with the Reconsolidation of Autobiographical Memories

**DOI:** 10.1371/journal.pone.0007519

**Published:** 2009-10-21

**Authors:** Lars Schwabe, Oliver T. Wolf

**Affiliations:** Department of Cognitive Psychology, Ruhr-University Bochum, Bochum, Germany; University of Queensland, Australia

## Abstract

It is commonly assumed that, with time, an initially labile memory is transformed into a permanent one via a process of consolidation. Yet, recent evidence indicates that memories can return to a fragile state again when reactivated, requiring a period of reconsolidation. In the study described here, we found that participants who memorized a story immediately after they had recalled neutral and emotional experiences from their past were impaired in their memory for the neutral (but not for the emotional) experiences one week later. The effect of learning the story depended critically on the preceding reactivation of the autobiographical memories since learning without reactivation had no effect. These results suggest that new learning impedes the reconsolidation of neutral autobiographical memories.

## Introduction

Memories are built in stages. Initially, novel information is acquired and retained for a short period. This transient short-term memory is transformed into a lasting memory via a consolidation process depending on protein synthesis [1]. For decades, it has been commonly assumed that consolidated memories are not subject to further modification. However, this view, has been challenged by animal studies suggesting that memories return to a fragile state when reactivated, making them susceptible to the same manipulations as the original consolidation process [2]–[4]. A time-limited process of reconsolidation appears to be necessary to render reactivated memories stable again [5], [6]. Corroborating the animal studies, recent studies in humans show that memories are altered when a beta-blocker or a new learning task is given after their reactivation [7]–[11].

Manipulating memory reconsolidation provides a unique opportunity to change unwanted, e.g. traumatic, memories in a favorable manner. Indeed, there is first evidence that the administration of a beta-blocker after the reactivation of traumatic memories may reduce emotional responding to the traumatic event in post traumatic stress disorder [12]. Interestingly, a recent rodent study suggested comparable effects after a drug-free intervention, namely, learning new information after memory reactivation [13]. Although there are human studies demonstrating altered memory when new material is learned after reactivation, these studies focused only on procedural memory [10], conditioning [7], [11] or the memory for a list of items learned in the laboratory [8], [9]. Whether these findings can be translated to memories for events people experienced in their “real” (i.e. every day) life is an important, but yet unanswered, question in reconsolidation research and its potential application in clinical, educational or legal settings.

Thus, the aim of the present experiment was to examine whether autobiographical memories can be modified by learning new episodic material after memories have been reactivated. Participants memorized an Indian folk tale (“The War of the Ghosts” by Bartlett [14]; interference task) after they had recalled neutral and emotional experiences from their past. In order to rule out non-specific effects of learning the tale, another group of participants learned the story without prior reactivation of autobiographical memories [15]. A third group recalled the personal experiences but did not learn the story afterwards; a fourth group did neither reactivate the autobiographical memories, nor learn the story. We hypothesized that learning new information after the reactivation of autobiographical memories would reduce the richness of these memories.

## Methods

### Participants and general procedure

Ninety-six healthy young adults (48 men, 48 women; age: M = 23.5 years, SEM = 0.3 years), all students of the Ruhr-University Bochum, received either course credits or a financial reward (15€) for participation in this experiment.

We used a 2 (reactivation vs. no reactivation) ×2 (interference vs. no interference) factorial between-subject design resulting in four experimental conditions: reactivation+interference, reactivation only, interference only, neither reactivation nor interference (control group). Groups differed mainly with respect to the experimental manipulation on day 1. Participants in the *reactivation+interference* group completed an autobiographical memory test (see below) and then learned an unfamiliar story. Participants in the *reactivation only* group completed the autobiographical memory test but did not learn the unfamiliar story. In contrast, participants in the *interference only* group did not perform the autobiographical memory test but learned only the unfamiliar story. Participants in the *control* group did not come to the laboratory on day 1.

One week later, participants in the *reactivation+interference* and *reactivation only* groups recalled the autobiographical events they had described the previous week. The *interference only* and *control* groups completed the autobiographical memory test as did the other two groups the week before. We randomly assigned 12 men and 12 women to each of the four groups.

### Memory reactivation

Autobiographical memories were reactivated by means of the autobiographical memory cueing test [16]. Participants were presented 2 positive (happy, interested), 2 neutral (busy, concentrated) and 2 negative (sad, angry) adjectives in randomized order (see [17]). They were instructed to remember, in as much detail as possible, one specific event from their own past for each adjective. Events should have occurred at least 24 hours and at maximum 2 weeks in the past. Participants were told that solely the specificity of the event was important, not its content. There was a time limit of 4 minutes for each of the 6 adjectives. After participants had written down the events, they were asked to indicate when each event had occurred and to give each memory a title (which should help to refer to the events on day 2).

In retrospect, participants described events that were negative, positive or neutral, according to the presented adjective. Examples of negative events were conflict with a friend or the death of a beloved person; examples of positive events were a nice party with friends or a successful exam; examples of neutral events were a certain lecture or the preparation for a test.

### Interference task

Immediately after the completion of the autobiographical memory test, participants of the *reactivation+interference* group read Bartlett's [14] “War of the Ghosts”, a complex story that was most likely to be clearly distinct from the participants' remembered events; participants in the *interference only* group read the story immediately after their arrival at the laboratory. They were given five minutes to memorize the story. Memory for the story was tested at the end of the experiment on day 2.

### Memory testing

The critical memory test took place one week after day 1. Participants in the *reactivation+interference* and *reactivation only* groups were presented the titles of the events they had described the week before and asked to remember as many details as possible of the referring event. In the *interference only* and *control* groups, participants completed the autobiographical memory test as did the other groups on day 1, except that they were instructed to remember events that were at least 1 week and at the most 3 weeks old. Again, participants had 4 minutes to describe the event associated with the title and adjective, respectively.

The autobiographical memories were assessed by two independent raters. One point was given for each detail (time, place, involved persons, weather, thoughts etc.) that was mentioned. The agreement between the two raters was very high (interrater reliability r_icc_ = 0.94). Discrepancies were discussed until an agreement was reached.

Points were first summed up for each event and then averaged for the positive, neutral and negative events.

### Mood assessment

To control for possible mood-congruent or mood-dependent memory effects [18], [19], we asked participants to complete the MDBF, a German multidimensional mood scale [20], at the beginning of the two experimental sessions (participants in the *control* group completed the MDBF on day 2 only). This questionnaire measures three dimensions of subjective feeling (“elevated vs. depressed mood”, “wakefulness vs. sleepiness”, “calmness vs. restlessness”) on a 5-point rating scale ranging from “not at all” ( = 1) to “very much” ( = 5).

## Results

### Age of the autobiographical memories

The age of the described memories was comparable in the four groups, *F*(3,92) = 1.63, *p* = .19, *η^2^* = 0.05. Events occurred, on average, 13.6 days (SEM = 0.5 days) before experimental day 2. Memories were narrated from the first person perspective suggesting that these reflected experiences of participants' own past.

### Effect of the interference task on the reconsolidation of autobiographical memories

Participants in the *reactivation only* and *reactivation+interference* groups did not differ in their memories on day 1 (12.8 vs. 12.4 details per event), *F*(1,46) = 0.10, *p* = .75, *η^2^*<0.01.

**Figure 1:**
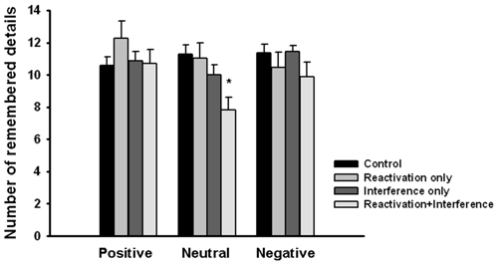
Number of details remembered of the positive, neutral and negative personal experiences on day 2. Neutral but not positive or negative autobiographical memories were reduced when participants learned new information after memory reactivation (* p<.01). Note that memories were about 2 weeks old when the memory test took place. Data represent means ± SEM.

Learning “The War of the Ghosts” immediately after the reactivation of the autobiographical memories reduced memory for the neutral but not for the emotional experiences one week later. As shown in Figure 1, participants in the *reactivation+interference* group remembered significantly less details of the neutral events than participants of the *reactivation only*, *interference only* or *control* groups whereas the memory for the positive and negative events remained unaffected by the interference task after reactivation. This is supported by a reactivation (yes vs. no) × interference (yes vs. no) × emotionality of the events (positive vs. neutral vs. negative) × sex (men vs. women) ANOVA which yielded a significant three-way interaction between reactivation, interference and emotionality of the events, *F*(2,174) = 5.02, *p*<.01, *η^2^* = 0.06. Follow-up ANOVAs indicated a significant reactivation × interference interaction for neutral, *F*(1,95) = 6.03, *p*<.02, *η^2^* = 0.06, but not for positive or negative events, both *F*(1,95)<1.36, both *p*>.25, both *η^2^*<0.02. Bonferroni-corrected post-hoc tests showed that memory for neutral events was significantly poorer in the *reactivation+interference* group relative to the other three groups, all *p*<.01.

Overall, women remembered more details than men, *F*(1,88) = 4.50, *p*<.04, *η^2^* = 0.05. Moreover, memories tended to be more detailed for positive than for negative and neutral experiences, *F*(2,182) = 2.95, *p*<.06, *η^2^* = 0.03. None of the other main or interaction effects reached statistical significance. The observed effect of the interference task after memory reactivation was not memory type specific (e.g. time, space, involved persons).

### Memory for the interference task

Participants in the *interference only* and *reactivation+interference* groups performed equally well in the memory test for “The War of the Ghosts”, *t*(46) = 1.11, *p* = .27. On average, they remembered 13.7 (SEM = 0.8) facts of the story.

### Mood

Mood effects cannot explain our results because groups had comparable MDBF scores on both experimental days, all *F*<1, all *p>.46*, *all η^2^*<0.03.

## Discussion

Retroactive interference by new learning shortly after the original learning is a well known phenomenon [21], [22]. Here, we show for the first time that new learning after the reactivation of personal experiences impairs the subsequent memory for these experiences, suggesting reconsolidation blockade in autobiographical memory.

Previous studies that demonstrated interference effects in human memory reconsolidation included solely neutral material [8]–[11]. In the present experiment, we examined memory for both neutral and emotional experiences and found reconsolidation effects for neutral but not for emotional material. How can the differential sensitivity of reactivated neutral and emotional memories to new learning be explained? Imagining positive or negative events is a common method to induce positive and negative mood states, respectively [23]. Thus, it appears likely that the retrieval of emotional memories altered the mood of the subjects. Neuromodulators, such as noradrenaline (which is known to play a crucial role in memory reconsolidation [3]), that were released during emotional memory retrieval may have affected the reconsolidation of neutral memories in particular, making the reconsolidation of these memories especially susceptible to the interfering influence of the rather disturbing “War of the Ghosts” story. Future studies should take this possibility into account and include subjective and objective (e.g. heart rate, salivary alpha amylase) measures of the arousal associated with the retrieval of the memories. Another potential explanation takes the strength of emotional memories into account. Emotionally arousing experiences are usually very well remembered [24]. This is because they activate brain structures (in particular the amygdala) and elicit the release of numerous hormones and neurotransmitters (e.g. adrenaline and noradrenaline) that facilitate memory consolidation [25]. Thus, emotional events were most likely better consolidated and therefore less sensitive to reconsolidation effects which are modulated by the strength of the memory [26]. While beta blockers are potent enough to disrupt the reconsolidation of emotional and traumatic memories [7], [12], this does not seem to be the case for learning of new information. Our findings suggest that reconsolidation-based approaches to “erasing” previous emotional or traumatic memories that rely solely on cognitive interference might not work because only neutral memories would be affected.

In addition to the emotional tone of the memories, reconsolidation effects depend on several other parameters. One important factor appears to be the age of the memories. In this study, memories were about 1 week old when they were reactivated. There is some evidence that memories become less vulnerable to reconsolidation effects the older they are [26], [27]. Hence, it can be speculated that memories from our more distant past are more likely to resist interfering influences when reactivated. Another factor relevant for memory reconsolidation is the reactivation context. It has recently been argued that new learning has to occur in the same spatial context as the original learning to modify the original memory trace [8]. In this study, however, memories were only 2 days old and there is evidence that memories become less context-dependent with age [28]. Our results suggest that travelling mentally back in time to recollect specific past episodes may be sufficient to render (autobiographical) memories susceptible to interference by new learning.

Memory reconsolidation has been viewed as a mechanism by which memories can be updated, either by the modification of the reactivated trace [29] or by the creation of a new version of the original trace [30]. Such an update, however, requires some degree of similarity between the original and the novel learning episode [9], [31]. In the present experiment, there was no obvious similarity between the recalled personal events and the Indian folk tale. There was also no indication of an updating of the autobiographical memories; none of the participants incorporated details of the folk tale into the recall of their own experiences. Our data suggest that memorizing the complex novel story claimed a considerable part of participants' limited cognitive processing capacities, leaving less cognitive resources for the reconsolidation of the reactivated memory. While the few previous studies that demonstrated reconsolidation effects in episodic memory [8], [9] showed that new learning did not reduce the number of correctly recalled items but led to a mixture of old and new items, the present findings provide the first evidence that reactivated autobiographical memories can degrade when they are not fully reconsolidated.

While reconsolidation is generally seen as an adaptive mechanism enabling the updating of reactivated memories in the light of new experiences [6], the results presented here point to its potential side effect: They suggest that reactivated autobiographical memories are, similar to new memories, in danger of being lost.
